# Outcomes of beta-blocker use in people living with chronic obstructive pulmonary disease and a co-existent beta-blocker indicated cardiovascular disease. Insights from a global federated network

**DOI:** 10.1186/s12890-026-04216-z

**Published:** 2026-03-04

**Authors:** Mert Kaşkal, Tommaso Bucci, Dennis Wat, Dilip Nazareth, Gregory Y. H. Lip, Freddy Frost

**Affiliations:** 1 Liverpool Centre for Cardiovascular Sciences, University of Liverpool, Liverpool John Moores University and Liverpool Heart and Chest Hospital, Liverpool, UK; 2 School of Medicine, Department of Medical Pharmacology, Marmara University, Istanbul, Turkey; 3 Department of Clinical Internal, Anesthesiologic and Cardiovascular Sciences, Sapienza University of Rome, Rome, Italy; 4 Adult Cystic Fibrosis Centre, Liverpool Heart and Chest Hospital NHS Foundation Trust, Liverpool, UK; 5 Danish Center for Health Services Research, Department of Clinical Medicine, Aalborg University, Aalborg University Hospital, Aalborg, Denmark; 6 Department of Cardiology, Lipidology and Internal Medicine with Intensive Coronary Care Unit, Medical University of Bialystok, Bialystok, Poland

**Keywords:** Beta-blockers, Chronic obstructive pulmonary disease, Cardiovascular disease

## Abstract

**Background:**

Beta-blockers (BBs) are a cornerstone of the management of cardiovascular diseases (CVD) such as heart failure with reduced ejection fraction (HFrEF), acute myocardial infarction (AMI), and atrial fibrillation (AF). Their use in patients with co-existing chronic obstructive pulmonary disease (COPD) remains controversial due to concerns about potential bronchoconstriction and respiratory side effects. This study aimed to assess the safety and effectiveness of BBs in patients with COPD and co-existing cardiovascular conditions using real-world data.

**Methods:**

We conducted a retrospective, propensity score–matched analysis using the TriNetX global federated research network. Patients with a diagnosis of both COPD and CVD (HFrEF, AMI, or AF) between January 2010 and January 2023 were included. Outcomes assessed over a one-year follow-up included all-cause mortality (primary outcome), emergency admissions (EA), and acute exacerbations of COPD (AECOPD). Subgroup analyses were conducted based on cardiovascular indication, BB selectivity, sex, and age group.

**Results:**

A total of 394,476 patients were included; 241,837 were BB users and 152,639 were non-users. After propensity score matching (*n* = 103,249 per group), there was no significant difference in mortality (HR: 0.98, 95% CI: 0.94–1.02). BB use was associated with an increased risk of EA (HR: 1.30, 95% CI: 1.22–1.40) and a modest increase in AECOPD (HR: 1.03, 95% CI: 1.02–1.04). Findings were consistent across subgroups.

**Conclusion:**

In people with COPD and a cardiac indication for BB use, the use of BBs was not associated with mortality benefit but was associated with a modest increased risk of AECOPD and a pronounced risk of increased EA.

**Clinical trial registration:**

Not applicable. This study is not a clinical trial; therefore, no trial registry, registration number, or registration date is required.

**Graphical abstract:**

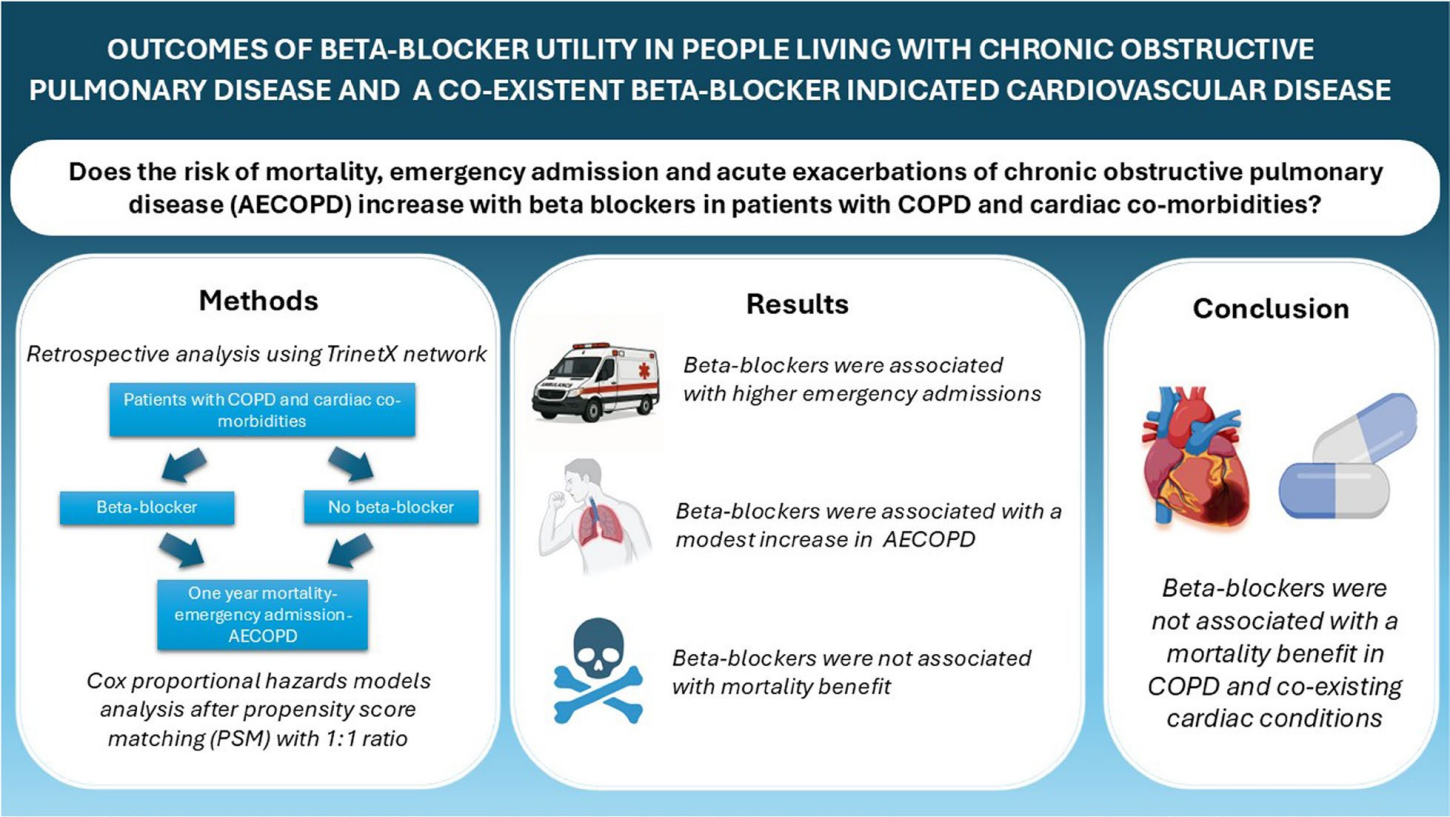

**Supplementary Information:**

The online version contains supplementary material available at 10.1186/s12890-026-04216-z.

## Key points

• Beta-blocker use does not significantly impact mortality in chronic obstructive pulmonary disease (COPD) patients with concomitant cardiovascular disease including heart failure with reduced ejection fraction, acute myocardial infarction, and atrial fibrillation.

• Beta-blocker use in COPD patients is associated with an increased risk of emergency admissions, even after adjustment for potential confounders.

• The elevated risk of emergency admissions in COPD patients using beta-blockers may be partially explained by the potential side effects associated with this drug class.

**Supplementary Information:**

The online version contains supplementary material available at 10.1186/s12890-026-04216-z.

## Introduction

Beta-blockers (BB) are widely recommended in the management of various cardiovascular conditions, including heart failure with reduced ejection fraction (HFrEF), acute myocardial infarction (AMI), and atrial fibrillation (AF), due to their proven benefits in reducing mortality and hospitalizations [1, 2]. However, their use in patients with chronic obstructive pulmonary disease (COPD) remains controversial, primarily because of the potential risk of bronchoconstriction [3].

Although some studies suggest that beta-blockers are generally safe in patients with COPD and cardiac comorbidities, clinical uncertainty and clinician hesitance persist, partly because most mortality data on beta-blockers come from trials that excluded patients with significant pulmonary disease, raising concerns about the applicability of these findings to the COPD population [4, 5].

In this study, we aimed to evaluate the safety and effectiveness of BB use in patients with COPD and specifically in cardiovascular conditions where beta-blockade is indicated for proven mortality benefit or other clinical benefit, such as HFrEF, AMI, and AF. By conducting a large-scale, propensity score-matched analysis using real-world data from the TriNetX network, we assessed one-year outcomes, including mortality, emergency admissions, and COPD exacerbations between BB users and non-users. We aimed to provide new insights for the benefit and risk of using BB in patients with COPD and co-existing cardiac diseases.

## Methods

### Study design

This study was designed as a retrospective observational analysis utilizing data from TriNetX, a global federated research network that uses electronic medical records from a wide range of healthcare institutions. These include academic medical centers, specialty clinics, and community hospitals, collectively representing health data from approximately 300 million individuals worldwide.

The available dataset within TriNetX includes information such as demographic characteristics, diagnostic codes (classified according to ICD-9-CM and ICD-10-CM standards), and prescription data, which are coded using Veteran Affairs (VA) medication codes. Additional information regarding the platform can be found at https://trinetx.com.

TriNetX adheres to the standards set by the Health Insurance Portability and Accountability Act (HIPAA) and complies with relevant U.S. federal regulations that protect the privacy and security of health information. All data used for analysis are de-identified in accordance with the HIPAA Privacy Rule. As TriNetX operates on a federated network model, analyses conducted using its platform do not require institutional ethical approval, as no identifiable patient-level data can be accessed. All methods were carried out in accordance with the ethical principles of the Declaration of Helsinki.

### Study cohort

The searches were performed on TriNetX platform. We compared baseline characteristics between BB users and non-users with cardiovascular disease and a pre-existing diagnosis of COPD. The cardiovascular diseases consist of the diagnosis of HFrEF, AMI and AF. For the HF subgroup, we included only patients with HFrEF, as BB have well-established evidence supporting their effectiveness in reducing mortality and hospitalizations in this population [6]. Patients with the above conditions were included if they were diagnosed between 1 January 2010 and 1 January 2023. BB use was defined as having at least one prescription recorded during the same period. Acute exacerbations of COPD (AECOPD) were identified using the ICD-10 code J44.1. Further details regarding the inclusion and exclusion criteria, as well as the ICD-10 codes used, are provided in Supplementary Table 1.

### Outcomes

Outcomes were assessed over a one-year follow-up period, defined as days 1 to 365 days following the diagnosis of COPD and the CVD. The primary outcome was the comparison of one-year all-cause mortality between BB users and non-users. Secondary outcomes included the incidence of emergency admissions (EA) and AECOPD within the same one-year period, comparing BB users to non-users. Additional sensitivity analyses were performed comparing BB users and non-users across subgroups, including patients prescribed selective and non-selective BBs, male and female patients, and elderly (≥ 65 years) and younger (< 65 years) individuals.

### Statistical analysis

Baseline characteristics between BB users and non-users were balanced using logistic regression and propensity score matching (PSM) at a 1:1 ratio. Matching was performed based on age, sex, race, the use of angiotensin-converting enzyme (ACE) inhibitors, angiotensin II receptor blockers (ARBs), antiarrhythmic agents, diuretics, and medications used for obstructive airway diseases. Additionally, patients were matched for the presence of essential hypertension, hyperlipidemia, diabetes mellitus, obesity, chronic kidney disease, neoplasms, HF, AF, AMI and cerebral infarction prior to the index event.

The balance of demographic and clinical variables between groups was evaluated using absolute standardized differences (ASD), with an ASD < 0.1 indicating adequate balance (Supplementary Fig. 1). These variables were selected based on their potential association with cardiovascular risk. Following matching, Cox proportional hazards models were used to calculate hazard ratios (HRs) and 95% confidence intervals (CIs) for the predefined outcomes, comparing BB users with non-users. Kaplan–Meier survival curves were constructed to illustrate differences in event-free survival between groups for both primary and secondary outcomes. Log-rank tests were used to assess between-group differences in the probability of experiencing the outcome over time.

All analyses were executed within the TriNetX platform, which utilises both R and Python for data analysis. The R Survival library v3.2-3 was used for survival analyses, while propensity risk scores were estimated using logistic regression, implemented via the scikit-learn package in Python version 3.7. All tests were two-tailed and statistical significance was defined as *p*-values < 0.05, indicating assuming a Type I error of less than 5% if the null hypothesis is true.

## Results

A total of 394,476 patients with COPD and concomitant cardiovascular disease (CVD) were included in the study. Among them, 241,837 patients were BB users (mean age: 71.3 ± 11.3 years; 44.2% female), while 152,639 were non-users (mean age: 73.9 ± 11.0 years; 46.7% female).

Before PSM, patients in the non-user group were older, more likely to be female, and had fewer comorbidities. Additionally, they were less frequently prescribed medications affecting the cardiovascular and pulmonary systems, such as ACE inhibitors, ARBs, anti-arrhythmic drugs, and inhaled therapies for obstructive airway diseases (e.g., corticosteroids and β2-agonists) (Table 1).

**Table 1:** Comparison of baseline characteristics between patients with COPD and CVD (HFrEF, AMI, AF) using and not using beta blockers, before and after propensity score matching

Baseline characteristics	Before propensity score matching			After propensity score matching		
	COPD-CVD BB use (*n*=241,837 )	COPD_CVD no BB use (*n*=152,639)	ASD	COPD-CVD BB use (*n*=103,249 )	COPD-CVD no BB use (*n*=103,249 )	ASD
Age, years (±SD)	71.3 ± 11.3	73.9 ± 11.0	0.234	73.1 ± 10.8	72.6 ± 11.3	0.050
Female, n (%)	106,892 (44.2)	71,282 (46.7)	0.043	47,804 (46.3)	48,011 (46.5)	0.009
White, n (%)	176,541 (73.0)	99,826 (65.4)	0.165	75,372 (73.0)	74,959 (72.6)	0.004
Obesity, n (%)	41,838 (17.3)	17,553 (11.5)	0.164	12,803 (12.4)	13,526 (13.1)	0.021
Arterial hypertension, n (%)	175,574 (72.6)	97,384 (63.8)	0.191	68,970 (66.8)	68,764 (66.6)	0.004
Hyperlipidemia, n (%)	133,978 (55.4)	57,240 (37.5)	0.365	46,565 (45.1)	47,288 (45.8)	0.013
Diabetes mellitus, n (%)	98,947 (40.9)	48,752 (31.9)	0.189	34,705 (33.6)	35,742 (34.6)	0.022
Chronic kidney failure, n (%)	78,596 (32.5)	34,825 (22.8)	0.285	25,628 (24.8)	26,633 (25.8)	0.022
Neoplasms, n (%)	71,618 (29.6)	41,710 (27.3)	0.050	29,134 (28.2)	29,134 (28.2)	0.001
Atrial fibrillation, n (%)	146,067 (60.4)	90,387 (59.2)	0.049	64,088 (62.1)	61,652 (59.7)	0.023
Acute myocardial infarction, n (%)	53,939 (22.3)	16,027 (10.5)	0.323	20,132 (19.5)	12,186 (11.8)	0.095
Heart failure, n (%)	114,833(47.3)	48,386 (31.7)	0.352	33,555 (32.5)	33,762 (32.7)	0.022
Cerebral infarction, n (%)	22,875 (9.5)	10,532 (6.9)	0.094	7,331 (7.1)	8,165 (7.9)	0.031
Drugs for obstructive air disease, n (%)	193,127 (79.9)	72,048 (47.2)	0.723	68,940 (66.7)	69,557 (67.4)	0.014
Antiarrythmics, n (%)	138,149 (57.1)	43,178 (28.3)	0.608	43,162 (41.8)	41,726 (40.4)	0.028
ACE inhibitors, n (%)	84,122 (34.8)	22,742 (14.9)	0.473	22,659 (21.9)	22,006 (21.3)	0.015
ARBs, n (%)	54,976 (22.7)	16,466 (10.8)	0.323	16,118 (15.6)	15,884 (15.4)	0.005
Diuretics, n (%)	159,983 (66.2)	49,106 (32.5)	0.716	49,628 (48.1)	48,135 (46.6)	0.029

*COPD* Chronic obstructive pulmonary disease, *HFrEF* Heart failure with reduced ejection fraction, *ACE* Angiotensin converting enzyme, *ARB* Angiotensin II receptor blockers, *BB* Beta-blockers, *ASD* Absolute standartized mean difference

Following PSM, 103,249 patients were matched in each group (Table 1). The median follow-up duration was 725 days (range: 124–1317 days) for BB users and 730 days (range: 130–1330 days) for non-users.

**Fig. 1:**
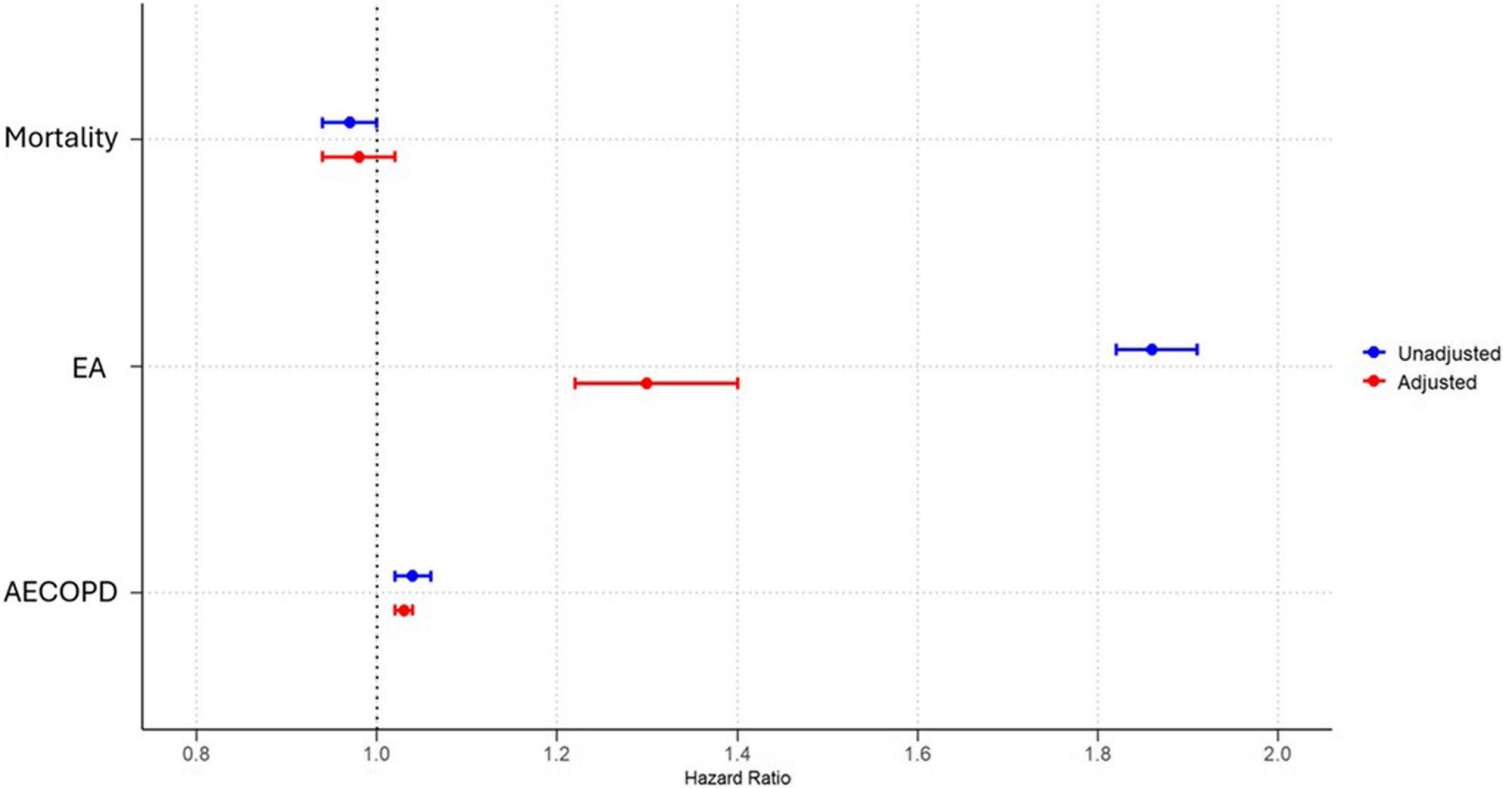
The risk of one year-mortality, emergency admission (EA) and acute exacerbation of chronic obstructive pulmonary disease (AECOPD) in patients with COPD and CVD (Heart failure reduced ejection fraction (HFrEF), acute myocardial infaction (AMI) and atrial fibrillation (AF) with beta-blocker use. Adjustments are made for age, sex, race, hypertension, diabetes mellitus, obesity, hyperlipidemia chronic kidney failure, cerebral infarction, neoplasms, HF, AF, AMI and drugs such as angiotensin converting enzyme inhibitors, angiotensin-II receptor blockers, anti-arrythmic drugs, diuretics, drugs used for obstructive airway disease, prior to index event with propensity score matching

Among 394,476 patients with COPD and CVD, the 1-year risk of mortality before PSM was similar between BB users and non-users (6.0% vs. 6.1%, HR: 0.97, 95% CI: 0.94–1.00; *p* = 0.06). The risk of EA was significantly higher in BB users compared to non-users (17.7% vs. 9.9%, HR: 1.86, 95% CI: 1.82–1.91; *p* < 0.0001), while AECOPD incidence was marginally increased (6.2% vs. 6.0%, HR: 1.04, 95% CI: 1.02–1.06; *p* = 0.03), (Table 2; Fig. 1).

**Table 2:** Risk of 1-year mortality, emergency admission and acute exacerbations of COPD versus beta-blocker users and non-users before and after propensity score matching

	Before Propensity Score Matching				After Propensity Score Matching			
	COPD-CVD and BB use (*n*=241,837)	COPD-CVD and no-BB use (*n*=152,639)	HR (95%CI)	*p*-value	COPD-CVD and BB use (*n*=103,249)	COPD-CVD and no-BB use (*n*=103,209)	HR (95%CI)	*p*-value
Risk of mortality (1-year) (n,%)	14,513 (6.0)	9,317 (6.1)	0.97 (0.94 to 1.00)	0.06	5,786 (5.6)	5,885 (5.7)	0.98 (0.94 to 1.02	0.08
Risk of EA (1-year) (n,%)	42,805 (17.7)	15,131 (9.9)	1.86 (1.82 to 1.91)	<0.0001	16,128 (15.6)	13,112 (12.7)	1.30 (1.22 to 1.40);	<0.0001
AECOPD incidence (1 year) (n,%)	14,993 (6.2)	9,158 (6.0)	1.04 (1.02 to 1.06)	0.03	6,510 (6.3)	6,289 (6.1)	1.03 (1.02 to 1.04);	0.02

	COPD-HFrEF and BB use (*n*=82,139)	COPD-HFrEF and no-BB use (*n*=24,606)	HR (95%CI)	p-value	COPD-HFrEF and BB use (*n*=19,402)	COPD-HFrEF and no-BB use (*n*=19,402)	HR (95%CI)	p-value
Risk of mortality (1-year) (n,%)	5,503 (6.7)	1,673 (6.8)	0.99 (0.94 to 1.05)	0.28	1,319 (6.8)	1,222 (6.3)	1.02 (0.95 to 1.07)	0.57
Risk of EA (1-year) (n,%)	16,674 (20.3)	2,731 (11.1)	1.76 (1.69 to 1.83)	<0.0001	3,434 (17.7)	2,445 (12.6)	1.36 (1.30 to 1.44)	<0.0001
AECOPD incidence (1 year) (n,%)	5,585 (6.8)	1,649 (6.7)	0.94 (0.86 to 1.02)	0.32	1,238 (6.3)	1,261 (6.5)	0.95 (0.88 to 1.04)	0.24

	COPD-AMI and BB use (*n*=52,420)	COPD-AMI and no-BB use (*n*=29,254)	HR (95%CI)	p-value	COPD-AMI and BB use (*n*=16,420)	COPD-AMI and no-BB use (*n*=16,420)	HR (95%CI)	p-value
Risk of mortality (1-year) (n,%)	2,831 (5.4)	1,521 (5.2)	1.03 (0.97 to 1.09)	0.22	788 (4.8)	837 (5.1)	0.99 (0.91 to 1.08)	0.94
Risk of EA (1-year) (n,%)	9,803 (18.7)	3,159 (10.8)	1.93 (1.86 to 2.00)	<0.0001	2,956 (18.0)	2,217 (13.5)	1.22 (1.16 to 1.28)	<0.0001
AECOPD incidence (1 year) (n,%)	3,774 (7.2)	2,136 (7.3)	0.98 (0.93 to 1.06)	0.53	1,199 (7.3)	1,182 (7.2)	1.00 (0.93 to 1.07)	0.72

	COPD-AF and BB use (*n*=165,142)	COPD-AF and no-BB use (*n*=96,792)	HR (95%CI)	p-value	COPD-AF and BB use (*n*=69,169)	COPD_AF and no-BB use (*n*=69,169)	HR (95%CI)	p-value
Risk of mortality (1-year) (n,%)	10,734 (6.5)	6,195 (6.4)	1.03 (0.99 to 1.07)	0.26	4,288 (6.2)	4,427 (6.4)	0.97 (0.91 to 1.06)	0.94
Risk of EA (1-year) (n,%)	26,588 (16.1)	8,905 (9.2)	1.87 (1.80 to 1.96)	<0.0001	10,455 (15.1)	4,703 (12.8)	1.34 (1.32 to 1.36)	<0.0001
AECOPD incidence (1 year) (n,%)	11,230 (6.8)	6,291 (6.5)	1.06 (1.04 to 1.09)	<0.0001	4,703 (6.8)	4,634 (6.7)	1.04 (1.02 to 1.07)	0.0002

After PSM, with 103,249 matched patients in the BB group and 103,209 in the non-user group, mortality remained comparable between the groups (5.6% vs. 5.7%, HR: 0.98, 95% CI: 0.94–1.02; *p* = 0.08). The elevated risk of EA persisted in BB users (15.6% vs. 12.7%, HR: 1.30, 95% CI: 1.22–1.40; *p* < 0.0001), and AECOPD incidence was marginally higher (6.3% vs. 6.1%, HR: 1.03, 95% CI: 1.02–1.04; *p* = 0.02) (Table 2; Fig. 1).

### Sensitivity analyses

To explore the robustness and generalizability of these findings we performed a number of ancillary subgroup and sensitivity analyses.

#### Chronic obstructive pulmonary disease and heart failure with reduced ejection fraction cohort

In the subgroup of patients with both COPD and HFrEF, the 1-year mortality risk was similar between BB users and non-users before PSM. The risk of EA was significantly higher among BB users compared to non-users (20.3% vs. 11.1%, HR: 1.76, 95% CI: 1.69–1.83; *p* < 0.0001), while AECOPD incidence was not found significant.

**Fig. 2:**
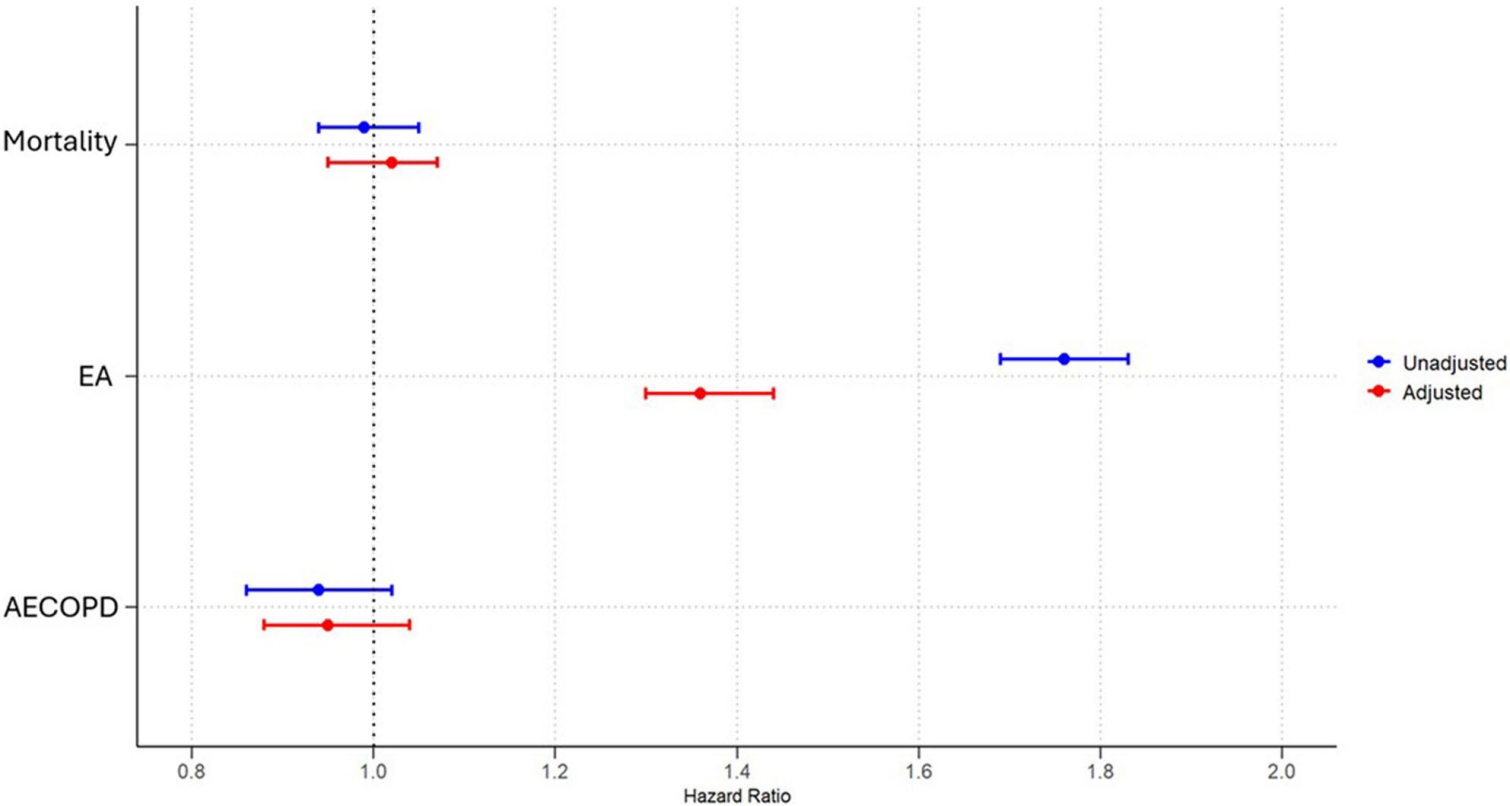
The risk of one year-mortality, emergency admission and chronic obstructive pulmonary disease (COPD) exacerbation in patients with COPD and heart failure reduced ejection fraction (HFrEF) with beta-blocker use after one year. Adjustments are made for age, sex, race, hypertension, diabetes mellitus, obesity, hyperlipidemia chronic kidney failure, cerebral infarction, neoplasms, AF, AMI and for drugs such as angiotensin converting enzyme inhibitors, angiotensin-II receptor blockers, anti-arrythmic drugs, diuretics, drugs used for obstructive airway disease, prior to index event with propensity score matching

After PSM, with 19,402 patients in each group, mortality between BB users and non-users was similar. The elevated risk of EA persisted post-matching (17.7% vs. 12.6%, HR: 1.36, 95% CI: 1.30–1.44; *p* < 0.0001), and AECOPD incidence remained non-significant (Table 2; Fig. 2). The characteristics of the HFrEF population are presented in Supplementary Table 2.

#### Chronic obstructive pulmonary disease and acute myocardial infarction cohort

Among patients with both COPD and AMI, the 1-year mortality risk was not significant between BB users and non-users before PSM. BB users had a significantly higher risk of EA compared to non-users (18.7% vs. 10.8%, HR: 1.93, 95% CI: 1.86–2.00; *p* < 0.0001), while AECOPD incidence was not significant between groups.

**Fig. 3:**
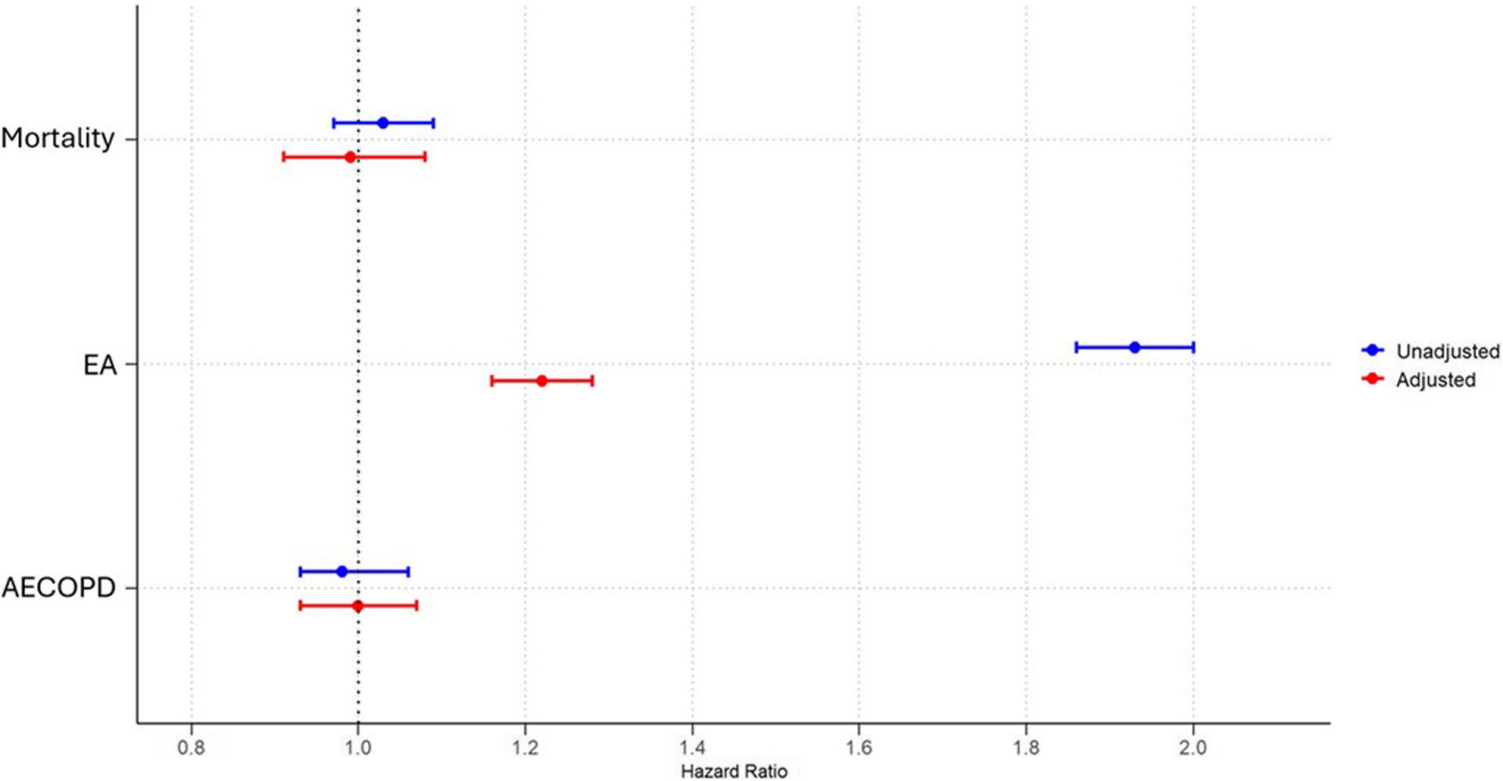
The risk of one year mortality, emergency admission and chronic obstructive pulmonary disease (COPD) exacerbation in patients with COPD and acute myocardial infarction (AMI) with beta-blocker (BB) use after one year. Adjustments are made for age, sex, race, hypertension, diabetes mellitus, obesity, hyperlipidemia chronic kidney failure, cerebral infarction, neoplasms, HF, AF and for drugs such as angiotensin converting enzyme inhibitors, angiotensin-II receptor blockers, anti-arrythmic drugs, diuretics, drugs used for obstructive airway disease, prior to index event with propensity score matching

After PSM, with 16,420 patients in each group, there was not significant difference in risk of mortality. The risk of EA remained elevated in BB users (18.0% vs. 13.5%, HR: 1.22, 95% CI: 1.16–1.28; *p* < 0.0001), whereas AECOPD incidence was similar (Table 2; Fig. 3). The characteristics of the AMI population are presented in Supplementary Table 3.

#### Chronic obstructive pulmonary disease and atrial fibrillation cohort

In patients with COPD and AF, the 1-year risk of mortality was similar between BB users and non-users prior to PSM. BB users had a significantly higher risk of EA compared to non-users (16.1% vs. 9.2%, HR: 1.87, 95% CI: 1.80–1.96; *p* < 0.0001), and a statistically significant increase in AECOPD incidence (6.8% vs. 6.5%, HR: 1.06, 95% CI: 1.04–1.09; *p* < 0.0001).

**Fig. 4:**
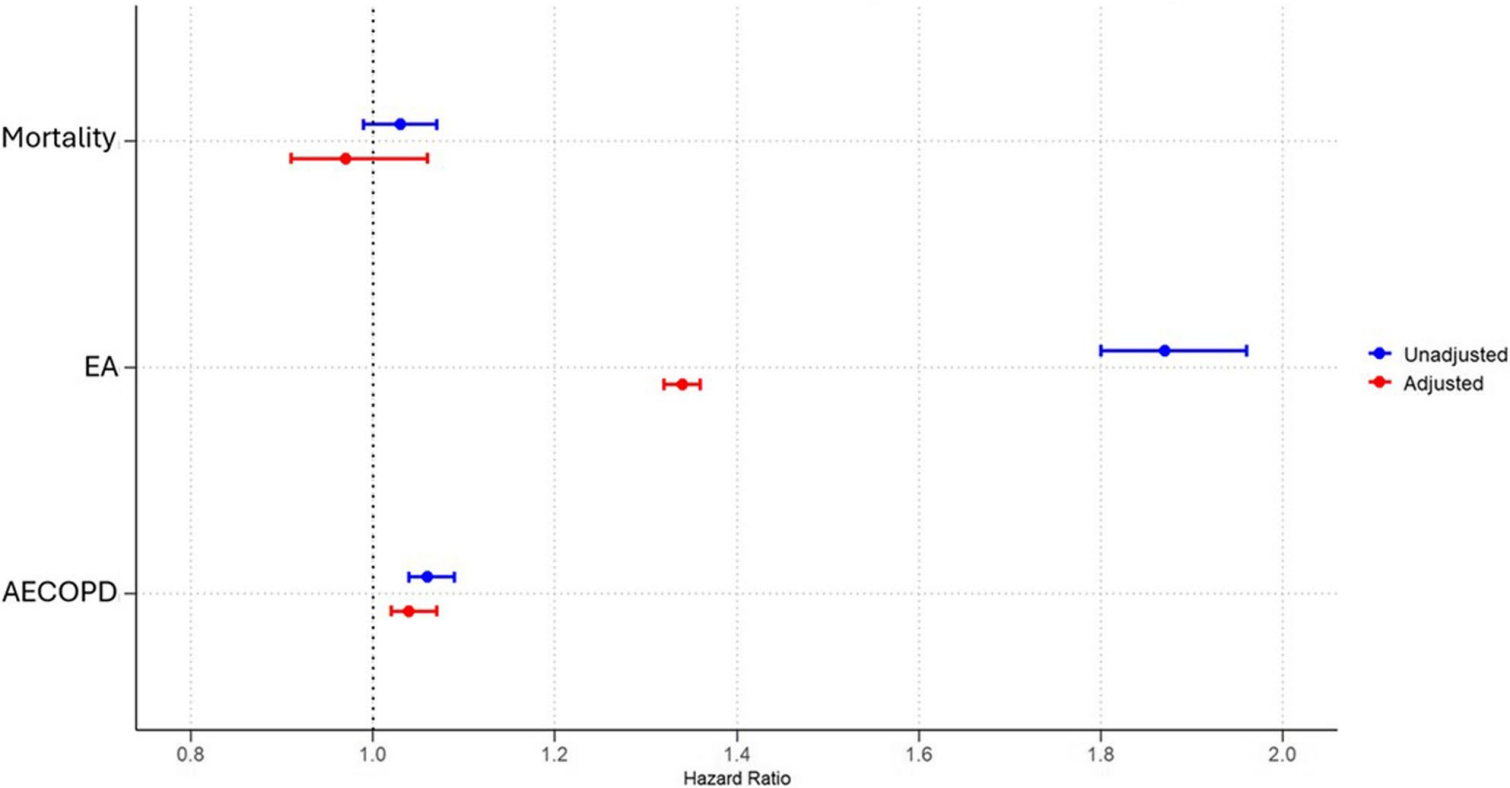
The risk of one year mortality, emergency admission and chronic obstructive pulmonary disease (COPD) exacerbation in patients with COPD and atrial fibrillation (AF) with beta-blocker (BB) use Adjustments are made for age, sex, race, hypertension, diabetes mellitus, obesity, hyperlipidemia chronic kidney failure, cerebral infarction, neoplasms, HF, AMI and for drugs such as angiotensin converting enzyme inhibitors, angiotensin-II receptor blockers, anti-arrythmic drugs, diuretics, drugs used for obstructive airway disease, prior to index event with propensity score matching

After PSM, with 69,169 patients in each group, mortality was non-significantly different between BB users and non-users. The elevated risk of EA persisted in BB users (15.1% vs. 12.8%, HR: 1.34, 95% CI: 1.32–1.36; *p* < 0.0001), and AECOPD incidence also remained slightly higher (6.8% vs. 6.7%, HR: 1.04, 95% CI: 1.02–1.07; *p* = 0.0002) (Table 2; Fig. 4). The characteristics of the AF population are presented in Supplementary Table 4.

### Subgroup analyses of beta-blocker use in chronic obstructive pulmonary disease and cardiovascular disease

#### Beta-blocker selectivity

In patients with COPD and CVD, the 1-year mortality risk was similar between users of selective BB (*n* = 206,039) and non-selective BB (*n* = 17,674) users prior to PSM. The risks of EA and AECOPD incidence were also not significant. After PSM, with 17,674 patients in each group, mortality remained similar as did the risks of EA and AECOPD indicating no significant difference in outcomes between selective and non-selective BB group (Supplementary Table 5).

#### Sex differences

In the male subgroup with COPD and CVD, the 1-year mortality risk was slightly lower in BB users compared to non-users before PSM (6.3% vs. 6.5%, HR: 0.95, 95% CI: 0.92–0.98; *p* = 0.002), and this difference was not statistically significant after PSM (6.3% vs. 6.0%, HR: 1.02, 95% CI: 0.98–1.06; *p* = 0.12). The risk of EA was significantly higher in BB users both before (16.2% vs. 9.1%, HR: 1.84, 95% CI: 1.79–1.89; *p* < 0.0001) and after PSM (14.2% vs. 11.4%, HR: 1.29, 95% CI: 1.26–1.33; *p* < 0.0001). AECOPD incidence was similar between the groups both before and after PSM (Supplementary Table 6).

Among female patients with COPD and CVD, the 1-year risk of mortality was similar between BB users and non-users before and after PSM. The risk of EA was significantly higher in BB users both before (18.0% vs. 11.6%, HR: 1.75, 95% CI: 1.71–1.79; *p* < 0.0001) and after PSM (16.3% vs. 13.5%, HR: 1.28, 95% CI: 1.24–1.32; *p* < 0.0001). AECOPD incidence showed no statistically significant difference between the groups before or after PSM (Supplementary Table 6).

#### Age

In older patients (age ≥ 65 years) with COPD and CVD, the 1-year mortality risk was non-significant between BB users and non-users before and after PSM. The risk of EA was significantly higher in BB users compared to non-users both before (15.8% vs. 9.1%, HR: 1.85, 95% CI: 1.81–1.89; *p* < 0.0001) and after PSM (14.5% vs. 11.6%, HR: 1.31, 95% CI: 1.26–1.33; *p* < 0.0001). AECOPD incidence was non-significant in BB users compared to non-users before and after matching (supplementary Table 7).

In younger patients (age < 65 years) with COPD and CVD, BB use was associated with a *significantly higher 1-year mortality risk* prior to PSM compared to non-users (3.8% vs. 3.1%, HR: 1.20, 95% CI: 1.12–1.28; *p* < 0.0001), though this difference was no longer significant after PSM. The risk of EA remained consistently higher in BB users both before (22.8% vs. 14.2%, HR: 1.48, 95% CI: 1.42–1.54; *p* < 0.0001) and after PSM (20.5% vs. 18.2%, HR: 1.17, 95% CI: 1.10–1.24; *p* < 0.0001). AECOPD incidence was similar between groups before and after matching (supplementary Table 7).

## Discussion

In this propensity score-matched retrospective analysis of patients with COPD and underlying cardiovascular comorbidities, our primary findings indicate no significant difference in 1-year mortality was found between BB users and non-users in patients with COPD and CVD. Second, an increased risk of EA among BB users compared to non-users in patients with COPD with concomitant CVD. Third, we also observed a modest increase in the incidence of AECOPD in BB users.

The management of patients with co-existing COPD and HFrEF presents a significant challenge due to the disease burden and high mortality rates in this population [7]. One of the primary concerns in treating these patients is the use of BBs. While BBs have been shown to significantly reduce hospitalization and mortality in patients with HFrEF, their use in individuals with concomitant COPD remains controversial due to the potential risk of bronchoconstriction [8, 9].

In our study, we observed an increased risk of EA and AECOPD in patients with COPD and HFrEF who were prescribed BBs. This finding may potentially be attributed to the bronchoconstrictive effects of BBs. Although selective BBs are commonly prescribed in this population, they may still exert effects on bronchial smooth muscle cells, potentially leading to bronchoconstriction [10]. Previous studies have suggested that BBs, particularly selective ones, may improve outcomes in patients with COPD, with some reports indicating a reduction in AECOPD rates [11, 12]. For instance, findings from the Bisoprolol in COPD Study (BICS) showed that treatment with bisoprolol did not increase the risk of COPD exacerbations requiring treatment with oral corticosteroids or antibiotics [13]. However, findings from the BLOCK-COPD trial (which was terminated early due to potential adverse safety signals) found that COPD patients receiving metoprolol, a selective BB, experienced increased rates of AECOPD aligning with the results observed in our study cohort [14].

Interestingly, we did not identify a significant mortality benefit between BB users and non-users in this population in contrast to prior studies which showed reduction in mortality rates in patients with HFrEF [15, 16]. However, the potential exclusion of patients with COPD in prior clinical trials may represent a limitation in assessing mortality among individuals with co-existing COPD and HFrEF. Our data suggests a careful risk-benefit assessment is essential when considering BB for patients with concomitant COPD and HFrEF to ensure that potential benefits outweigh the risks associated with bronchoconstriction.

BBs have well-established benefits in the management of patients following AMI especially in studies performed in the pre-reperfusion era, including reducing mortality, preventing arrhythmias, and improving long-term cardiac function with minimal adverse respiratory effects [17, 18]. Contrary to the findings in the existing literature, we found that BB use was associated with an increased risk of EA in the AMI cohort [19, 20]. This association may be again explained by the potential bronchoconstrictive effects of BB, which could contribute to the increased rate related emergency visits. Additionally, we do not have data on the severity of COPD diagnosis in our study population, so the cohort may be skewed toward more severe cases—similar to the before mentioned BLOCK-COPD study [14] —which reported an increased rate of AECOPD with metoprolol use.

Overall evidence for BB benefits post-AMI in the reperfusion era are less certain, and the generalizability of these findings to COPD, which is often an exclusion criteria in clinical trials is challenging [15, 21]. A further consideration is that our findings, and the lack of AMI mortality benefit for BB in the reperfusion era may triangulate with the fact in people with COPD, MI diagnosis may be delayed and hence successful timely reperfusion rates may be lower [22]. People with COPD may therefore be more generalizable to the pre-reperfusion era evidence base [23]. This is speculation but the relative dearth of information about COPD people in CVD trials means we cannot exclude this as a potential explanation.

BBs are among the first-line agents for managing AF, as they have been shown to reduce AF recurrence and help maintain sinus rhythm [24]. However, in patients with coexisting COPD and AF, their use is often approached with caution due to concerns about potential adverse respiratory effects. The coexistence of AF and COPD is associated with increased disease burden and worse clinical outcomes in this patient population [25]. Due to safety concerns, BBs are less frequently prescribed in patients with both COPD and AF [25, 26]. Additionally, several studies have reported that BB therapy is not associated with adverse outcomes in this subgroup, supporting the safety of their use [25, 27].

In contrast to the existing literature, our study found an increased risk of EA and slight increase in AECOPD in patients with concomitant COPD and AF who were treated with BBs, without evidence of a mortality benefit. Supporting this in a recent study by Liang et al. found no mortality benefit with BBs in patients with concomitant AF and COPD [28]. Potential explanations for these findings may include the possibility the use of BBs could not be sufficient to control the symptoms of AF in these patients potentially leading to an increased number of EA. In contrast, cardioselective calcium channel blockers such as verapamil may be more effective for the prevention of AF in these patients [29]. Overall, these findings suggest that a more cautious approach should be taken when prescribing BBs in this population—similar to the careful consideration required in patients with HFrEF and AMI—due to the potential for increased adverse effects without clear benefit.

We found no difference in the outcomes between selective and non-selective BBs in sensitivity analysis. These findings suggest that BB selectivity does not significantly influence short-term clinical outcomes in this population. In contrast, prior observational studies have reported an increased risk of AECOPD associated with non-selective BBs, leading to recommendations that selective BBs be more preferred in this population [20, 30]. However, our results did not support a differential impact based on BB selectivity. When we conducted further sensitivity analyses stratified by sex, we observed an increased risk of EA in both male and female patients, while no significant differences were found in mortality or acute exacerbations of AECOPD consistent with the findings from our main cohort. To our knowledge, no previous study has rigorously examined the interaction between sex and BB use in relation to COPD outcomes. Our findings suggest that sex does not significantly modify the association between BB use and clinical outcomes in patients with COPD and concomitant CVD.

### Limitations

The retrospective and observational nature of the study makes it susceptible to selection bias, indication bias and other unmeasured confounders. In the PSM analysis, we balanced the two populations based on the prevalence of cardiovascular disease, but not its severity or specific type. This may have led to residual differences in baseline risk, which could have influenced the risk of incident AECOPD. Modest increase found in the incidence of AECOPD was observed after BB use (HR:1.03), the statistically significant post-matching effect size was small and may reflect residual confounding or outcome misclassification. Our analysis was based on inpatient data, which included all hospital admissions for individuals in the cohort. As a result, we were unable to determine whether hospitalizations were specifically related to cardiac or pulmonary complications, limiting the specificity of our outcome assessment. Furthermore, our study relied on ICD-10 diagnostic codes, which do not provide detailed information on disease severity. Therefore, we could not account for the clinical severity of COPD, AF, AMI, or HFrEF in our analysis. Also important clinical measures of COPD severity, including lung function parameters and COPD Assessment Test (CAT) scores, history of COPD exacerbations were not assessed which is also a limitation. As this study was specifically designed to evaluate the effects of BBs inhaled COPD therapies were not examined; therefore, potential confounding related to COPD pharmacotherapy and disease severity should be considered when interpreting our findings. Emergency admissions were captured as an all-cause endpoint without differentiation by respiratory, cardiovascular, or other causes. This limits interpretability and precludes definitive causal inference regarding the respiratory safety of BB use. Lastly, data on medication adherence were not available. Variability in patient adherence to BB therapy may have influenced the observed outcomes and should be considered a potential limitation.

## Conclusion

In people with COPD and a cardiac indication for BB use, the use of BBs was not associated with mortality benefit but was associated with a modest increased risk of AECOPD and a pronounced risk of increased EA.

## Supplementary Information

Supplementary Material 1.

Supplementary Material 2.

Supplementary Material 3.

Supplementary Material 4.

Supplementary Material 5.

Supplementary Material 6.

Supplementary Material 7.

Supplementary Material 8.

## Data Availability

The datasets used and/or analysed during the current study are available from the corresponding author on reasonable request.
