# Supplementary material for: Outcomes of beta-blocker use in people living with chronic obstructive pulmonary disease and a co-existent beta-blocker indicated cardiovascular disease. Insights from a global federated network
Source: BMC Pulm Med. 2026 Mar 4;26:166. doi: 10.1186/s12890-026-04216-z (PMC13067551; doi:10.1186/s12890-026-04216-z)
Supplement: Supplementary file 1 — Supplementary Material 1. [file 12890_2026_4216_MOESM1_ESM.docx]

| **Supplementary Table 1.** ICD-10-CM and ATC codes for inclusion and exclusion criterias for the study cohorts | |
| --- | --- |
| **Patients with chronic obstructive pulmonary disease and heart failure with reduced ejection fraction and beta-blocker use** | |
| Inclusion Criteria | 1. Chronic Obstructive Pulmonary Disease (ICD-10-CM J44) between 1st January 2010 to 1st January 2024 |
| After Chronic Obstructive Pulmonary Disease Diagnosis | |
| Inclusion Criteria | 1. Systolic heart failure (ICD-10-CM I50.2) 2. Beta-blocking agents (ATC-C07) |
| Exclusion Criteria | 1. Unspecified diastolic heart failure (ICD-10-CM I50.3) |
| **Patients with chronic obstructive pulmonary disease and heart failure with reduced ejection fraction and no-beta-blocker use** | |
| Inclusion Criteria | 1. Chronic Obstructive Pulmonary Disease (ICD-10-CM J44) between 1st January 2010 to 1st January 2024 |
| After Chronic Obstructive Pulmonary Disease Diagnosis | |
| Inclusion Criteria | 1. Systolic heart failure (ICD-10-CM I50.2) |
| Exclusion Criteria | 1. Unspecified diastolic heart failure (ICD-10-CM I50.3) 2. Beta-blocking agents (ATC-C07) |
| **Patients with chronic obstructive pulmonary disease and acute myocardial infarction and beta-blocker use** | |
| Inclusion Criteria | 1. Chronic Obstructive Pulmonary Disease (ICD-10-CM J44) between 1st January 2010 to 1st January 2024 |
| After Chronic Obstructive Pulmonary Disease Diagnosis | |
| Inclusion Criteria | 1. Acute myocardial infarction (ICD-10-CM I20) 2. Beta-blocking agents (ATC-C07) |
| **Patients with chronic obstructive pulmonary disease and acute myocardial infarction and no beta-blocker use** | |
| Inclusion Criteria | 1. Chronic Obstructive Pulmonary Disease (ICD-10-CM J44) between 1st January 2010 to 1st January 2024 |
| After Chronic Obstructive Pulmonary Disease Diagnosis | |
| Inclusion Criteria | 1. Acute myocardial infarction (ICD-10-CM I20) |
| Exclusion Criteria | 1. Beta-blocking agents (ATC-C07) |
| **Patients with chronic obstructive pulmonary disease and atrial fibrillation and beta-blocker use** | |
| Inclusion Criteria | 1. Chronic Obstructive Pulmonary Disease (ICD-10-CM J44) between 1st January 2010 to 1st January 2024 |
| After Chronic Obstructive Pulmonary Disease Diagnosis | |
| Inclusion Criteria | 1. Unspecified atrial fibrillation (ICD-10-CM I48.91) 2. Beta-blocking agents (ATC-C07) |
| **Patients with chronic obstructive pulmonary disease and atrial fibrillation and no beta-blocker use** | |
| Inclusion Criteria | 1. Chronic Obstructive Pulmonary Disease (ICD-10-CM J44) between 1st January 2010 to 1st January 2024 |
| After Chronic Obstructive Pulmonary Disease Diagnosis | |
| Inclusion Criteria | 1. Unspecified atrial fibrillation (ICD-10-CM I48.91) |
| Exclusion Criteria | 1. Beta-blocking agents (ATC-C07) |
