# Supplementary material for: Outcomes of beta-blocker use in people living with chronic obstructive pulmonary disease and a co-existent beta-blocker indicated cardiovascular disease. Insights from a global federated network
Source: BMC Pulm Med. 2026 Mar 4;26:166. doi: 10.1186/s12890-026-04216-z (PMC13067551; doi:10.1186/s12890-026-04216-z)
Supplement: Supplementary file 2 — Supplementary Material 2. [file 12890_2026_4216_MOESM2_ESM.docx]

| **Baseline characteristics** | **Before propensity score matching** | | | **After propensity score matching** | | |
| --- | --- | --- | --- | --- | --- | --- |
|  | **COPD_HFrEF BB use**  **(n=82,139)** | **COPD_HFrEF no BB use**  **(n=24,606)** | **ASD** | **COPD_HFrEF BB use**  **(n=19,402)** | **COPD_HFrEF**  **no BB use**  **(n=19,402)** | **ASD** |
| **Age, years (±SD)** | 69.1 ± 11.8 | 71.6 ± 12.1 | 0.083 | 71.1 ± 11.4 | 70.5 ± 12.3 | 0.001 |
| **Female, n (%)** | 31,788 (38.7) | 9,646 (39.2) | 0.014 | 7,528 (38.8) | 7,547 (38.9) | 0.013 |
| **White, n (%)** | 55,197 (67.2) | 15,600 (63.4) | 0.032 | 12,922 (66.6) | 12,553 (64.7) | 0.023 |
| **Obesity, n (%)** | 16,017 (19.5) | 3,642 (14.8) | 0.130 | 2,949 (15.2) | 2,891 (14.9) | 0.003 |
| **Arterial hypertension, n (%)** | 58,843 (71.2) | 15,871 (64.5) | 0.195 | 12,767 (65.8) | 12,514 (64.5) | 0.009 |
| **Hyperlipidemia, n (%)** | 49,776 (60.6) | 12,131 (49.3) | 0.042 | 9,934 (51.2) | 9,856 (50.8) | 0.008 |
| **Diabetes mellitus, n (%)** | 38,852 (47.3) | 11,220 (45.6) | 0.046 | 8,964 (46.2) | 8,886 (45.8) | 0.008 |
| **Chronic kidney failure, n (%)** | 28,338 (34.5) | 7,283 (29.6) | 0.065 | 6,247 (32.2) | 6,073 (31.3) | 0.011 |
| **Neoplasms, n (%)** | 21,614 (26.8) | 6,348 (25.8) | 0.010 | 5,064 (26.1) | 4,967 (25.6) | 0.006 |
| **Atrial fibrillation, n (%)** | 38,112 (46.4) | 10,384 (42.2) | 0.086 | 8,983 (46.3) | 8,944 (46.1) | 0.007 |
| **Acute myocardial infarction, n (%)** | 22,670 (27.6) | 5,536 (22.5) | 0.071 | 4,424 (22.8) | 4,346 (22.4) | 0.002 |
| **Cerebral infarction, n (%)** | 7,885 (9.6) | 1,821 (7.4) | 0.029 | 1,513 (7.8) | 1,475 (7.6) | 0.001 |
| **Drugs for obstructive air disease, n (%)** | 68,823 (83.8) | 13,583 (55.2) | 0.123 | 12,708 (65.5) | 13,155 (67.8) | 0.017 |
| **Antiarrythmics, n (%)** | 51,255 (62.4) | 10,900 (44.3) | 0.247 | 8,440 (43.5) | 8,110 (41.8) | 0.014 |
| **ACE inhibitors, n (%)** | 31,127 (45.2) | 6,767 (27.5) | 0.314 | 5,180 (26.7) | 4,928 (25.4) | 0.021 |
| **ARBs, n (%)** | 20,535 (25.0) | 3,912 (15.9) | 0.193 | 4,598 (23.7) | 4,676 (24.1) | 0.009 |
| **Diuretics, n (%)** | 66,861 (81.4) | 11,442 (46.5) | 0.742 | 11,544 (59.5) | 11,428 (58.9) | 0.007 |

**Supplementary table 2. Comparison of baseline characteristics between patients with COPD and HFrEF using and not using beta blockers, before and after propensity score matching.**

COPD: Chronic obstructive pulmonary disease, HFrEF: Heart failure with reduced ejection fraction ACE: Angiotensin converting enzyme, ARB: Angiotensin II receptor blockers, BB: Beta-blockers ASD: Absolute standartized mean difference
