# Supplementary material for: Outcomes of beta-blocker use in people living with chronic obstructive pulmonary disease and a co-existent beta-blocker indicated cardiovascular disease. Insights from a global federated network
Source: BMC Pulm Med. 2026 Mar 4;26:166. doi: 10.1186/s12890-026-04216-z (PMC13067551; doi:10.1186/s12890-026-04216-z)
Supplement: Supplementary file 3 — Supplementary Material 3. [file 12890_2026_4216_MOESM3_ESM.docx]

| **Baseline Characteristics** | **Before propensity score matching** | | | **After propensity score matching** | | |
| --- | --- | --- | --- | --- | --- | --- |
|  | **COPD_AMI**  **BB use**  **(n= 52,420)** | **COPD_AMI**  **no BB use**  **(n=29,254)** | **ASD** | **COPD_AMI**  **BB use**  **(n=16,420)** | **COPD_AMI**  **no BB use**  **(n=16,420)** | **ASD** |
| **Age, years (±SD)** | 69.3 ± 11.5 | 70.6 ± 11.6 | 0.107 | 70.0 ± 11.3 | 69.6 ± 11.9 | 0.030 |
| **Female, n (%)** | 24,218 (46.2) | 14,363 (49.1) | 0.057 | 7,668 (46.7) | 7,685 (46.8) | 0.002 |
| **White, n (%)** | 37633 (71.8) | 17,610 (60.2) | 0.235 | 11510 (70.1) | 11,411 (69.5) | 0.013 |
| **Arterial hypertension, n (%)** | 55,471 (77.2) | 17,138 (66.1) | 0.249 | 13,244 (67.6) | 13,375 (68.2) | 0.014 |
| **Hyperlipidemia, n (%)** | 46,402 (64.5) | 11,571 (44.6) | 0.409 | 9,908 (50.6) | 10,148 (51.8) | 0.024 |
| **Diabetes mellitus, n (%)** | 34,068 (47.4) | 9,562 (36.8) | 0.215 | 7,345 (37.5) | 7,623 (38.9) | 0.029 |
| **Chronic kidney failure, n (%)** | 27,316 (38.0) | 6,693 (25.8) | 0.264 | 5,265 (26.8) | 5,562 (28.3) | 0.034 |
| **Neoplasms, n (%)** | 20,964 (29.2) | 7,056 (27.2) | 0.044 | 5,405 (27.6) | 5,314 (27.1) | 0.010 |
| **Obesity, n (%)** | 15,165 (21.1) | 3,508 (13.5) | 0.201 | 2,819 (14.4) | 2,904 (14.8) | 0.012 |
| **Atrial fibrillation, n (%)** | 24,792 (34.5) | 6,027 (23.2) | 0.251 | 5,724 (29.2) | 4,624 (26.6) | 0.082 |
| **Heart failure, n (%)** | 31,137 (59.4) | 11,467 (39.2) | 0.411 | 6,896 (42.0) | 7,126 (43.4) | 0.028 |
| **Cerebral infarction, n (%)** | 8,010 (11.1) | 8,123 (8.1) | 0.103 | 1,538 (7.8) | 1,718 (8.7) | 0.033 |
| **Drugs for obstructive air disease, n (%)** | 59,654 (83.0) | 12,983 (50.1) | 0.746 | 12,733 (65.1) | 12,909 (65.9) | 0.018 |
| **Antiarrythmics, n (%)** | 45,052 (62.7) | 7,959 (30.7) | 0.678 | 8,159 (41.6) | 7,919 (40.4) | 0.024 |
| **ACE inhibitors, n (%)** | 29,951 (41.2) | 4,523 (17.4) | 0.540 | 4,675 (23.9) | 4,511 (23.0) | 0.019 |
| **ARBs, n (%)** | 17,282 (24.5) | 2,718 (10.5) | 0.285 | 2,298 (14.0) | 2.249 (13.7) | 0.004 |
| **Diuretics, n (%)** | 48,644 (67.7) | 8,267 (31.9) | 0.768 | 8,483 (43.3) | 8,246 (42.1) | 0.024 |

**Supplemantary table 3. Comparison of baseline characteristics between patients with COPD and AMI using and not using beta blockers, before and after propensity score matching.**

COPD: Chronic obstructive pulmonary disease, AMI: Acute myocardial infarction, ACE: Angiotensin converting enzyme, ARB: Angiotensin II receptor blockers, BB: Beta-blockers ASD: Absolute standartized mean difference
