# Supplementary material for: Outcomes of beta-blocker use in people living with chronic obstructive pulmonary disease and a co-existent beta-blocker indicated cardiovascular disease. Insights from a global federated network
Source: BMC Pulm Med. 2026 Mar 4;26:166. doi: 10.1186/s12890-026-04216-z (PMC13067551; doi:10.1186/s12890-026-04216-z)
Supplement: Supplementary file 4 — Supplementary Material 4. [file 12890_2026_4216_MOESM4_ESM.docx]

| **Baseline characteristics** | **Before propensity score matching** | | | **After propensity score matching** | | |
| --- | --- | --- | --- | --- | --- | --- |
|  | **COPD_AF**  **BB use**  **(n=165,142)** | **COPD_AF**  **no BB use**  **(n=96,792)** | **ASD** | **COPD_AF**  **BB use**  **(n=69,169)** | **COPD_AF**  **no BB use**  **(n=69,169)** | **ASD** |
| **Age, years (±SD)** | 73.2 ± 10.6 | 75.2 ± 10.3 | 0.191 | 74.8 ± 10.1 | 74.2 ± 10.6 | 0.052 |
| **Female, n (%)** | 76,130 (46.1) | 45,874 (47.4) | 0.029 | 33,132 (47.9) | 32,994 (47.7) | 0.006 |
| **White, n (%)** | 124,682 (75.5) | 64,463 (66.6) | 0.119 | 51,946 (75.1) | 51,600 (74.6) | 0.009 |
| **Arterial hypertension, n (%)** | 123,691 (74.9) | 63,689 (65.8) | 0.200 | 47,796 (69.1) | 47,519 (68.7) | 0.009 |
| **Hyperlipidemia, n (%)** | 93,966 (56.9) | 37,555 (38.8) | 0.368 | 32,094 (46.4) | 32,509 (47.0) | 0.011 |
| **Diabetes mellitus, n (%)** | 68,369 (41.4) | 31,167 (32.2) | 0.128 | 23,379 (33.8) | 24,410 (34.9) | 0.050 |
| **Chronic kidney failure, n (%)** | 57,469 (34.8) | 23,327 (24.1) | 0.237 | 18,261 (26.4) | 18,883 (27.3) | 0.022 |
| **Neoplasms, n (%)** | 53,671 (32.5) | 27,876 (28.8) | 0.082 | 21,097 (30.5) | 20,889 (30.2) | 0.007 |
| **Obesity, n (%)** | 32,533 (19.7) | 12,196 (12.6) | 0.193 | 9,614 (13.9) | 10,099 (14.6) | 0.018 |
| **Acute myocardial infarction, n (%)** | 25,102 (15.2) | 6,582 (6.8) | 0.272 | 6,364 (9.2) | 5,188 (7.5) | 0.091 |
| **Heart failure, n (%)** | 44,919 (27.2) | 19,455 (20.1) | 0.184 | 15,563 (22.5) | 15,079 (21.8) | 0.043 |
| **Cerebral infarction, n (%)** | 17,010 (10.3) | 7,066 (7.3) | 0.104 | 5,395 (7.8) | 5,879 (8.5) | 0.027 |
| **Drugs for obstructive air disease, n (%)** | 133,435 (80.8) | 45,202 (46.7) | 0.758 | 46,482 (67.2) | 47,312 (68.4) | 0.025 |
| **Antiarrythmics, n (%)** | 98,590 (59.7) | 28,070 (29.0) | 0.651 | 30,158 (43.6) | 29,189 (42.2) | 0.029 |
| **ACE inhibitors, n (%)** | 55,488 (33.6) | 14,132 (14.6) | 0.454 | 15,286 (22.1) | 14,802 (21.4) | 0.014 |
| **ARBs, n (%)** | 48,953 (23.2) | 14,977 (11.1) | 0.326 | 14,806 (16.2) | 14,709 (16.1) | 0.003 |
| **Diuretics, n (%)** | 145,583 (69.2) | 45,590 (33.9) | 0.756 | 46,845 (51.4) | 45,273 (49.7) | 0.035 |

**Supplementary table 4. Comparison of baseline characteristics between patients with COPD and AF using and not using beta blockers, before and after propensity** score matching.

COPD: Chronic obstructive pulmonary disease, AF: Atrial fibrillation, ACE: Angiotensin converting enzyme, ARB: Angiotensin II receptor blockers, BB: Beta-blockers ASD: Absolute standartized mean difference
