# Supplementary material for: Outcomes of beta-blocker use in people living with chronic obstructive pulmonary disease and a co-existent beta-blocker indicated cardiovascular disease. Insights from a global federated network
Source: BMC Pulm Med. 2026 Mar 4;26:166. doi: 10.1186/s12890-026-04216-z (PMC13067551; doi:10.1186/s12890-026-04216-z)
Supplement: Supplementary file 5 — Supplementary Material 5. [file 12890_2026_4216_MOESM5_ESM.docx]

| **Supplementary table 5. Risk of 1-year mortality, emergency admission and acute exacerbations of COPD versus selective and non-selective beta-blockers** | | | | | | | | |
| --- | --- | --- | --- | --- | --- | --- | --- | --- |
|  | **Before Propensity Score Matching** | | | | **After Propensity Score Matching** | | | |
|  | **COPD-CVD and sBB use**  **(n=206,039)** | **COPD-CVD and nBB use**  **(n=17,674)** | **HR (95%CI)** | **p-value** | **COPD-CVD**  **and sBB use**  **(n=17,674)** | **COPD-CVD**  **and nBB use**  **(n=17,674)** | **HR (95%CI)** | **p-value** |
| **Risk of mortality**  **(1-year) n (%)** | 12,568 (6.1) | 1,131 (6.4) | 0.98 (0.92 to 1.04) | 0.64 | 1,113 (6.3) | 1,149 (6.5) | 1.01 (0.93 to 1.09) | 0.73 |
| **Risk of EA**  **(1-year) n (%)** | 33,584 (16.3) | 3,111 (17.6) | 0.99 (0.96 to 1.02) | 0.66 | 2,987 (16.9) | 3,093 (17.5) | 1.02 (0.96 to 1.08) | 0.66 |
| **AECOPD incidence**  **(1 year) n (%)** | 12,362 (6.0) | 1,078 (6.1) | 1.04 (0.98 to 1.10) | 0.43 | 1,091 (6.2) | 1,082 (6.1) | 1.06 (0.96 to 1.16) | 0.10 |
| **HR:Hazard Ratio, CI: Confidence Interval, EA: Emergency admission, COPD: Chronic obstructive pulmonary disease, AECOPD: acute exacerbation of COPD HFrEF: Heart failure reduced ejection fraction, AMI: Acute myocardial infarction, AF: Atrial fibrillation, sBB: Selective beta-blockers, nBB: Non-selective beta-blockers** | | | | | | | | |
