# Supplementary material for: Outcomes of beta-blocker use in people living with chronic obstructive pulmonary disease and a co-existent beta-blocker indicated cardiovascular disease. Insights from a global federated network
Source: BMC Pulm Med. 2026 Mar 4;26:166. doi: 10.1186/s12890-026-04216-z (PMC13067551; doi:10.1186/s12890-026-04216-z)
Supplement: Supplementary file 6 — Supplementary Material 6. [file 12890_2026_4216_MOESM6_ESM.docx]

| **Supplementary table 6. Risk of 1-year mortality, emergency admission and acute exacerbations of COPD versus beta-blocker users and non-users based on sex before and after propensity score matching** | | | | | | | | |
| --- | --- | --- | --- | --- | --- | --- | --- | --- |
| **Male patients** | **Before Propensity Score Matching** | | | | **After Propensity Score Matching** | | | |
|  | **COPD-CVD and BB use**  **(n=151,074)** | **COPD-CVD and no-BB use**  **(n=88,990)** | **HR (95%CI)** | **p-value** | **COPD-CVD**  **and BB use**  **(n=60,615)** | **COPD-CVD**  **and no-BB use**  **(n=60,615)** | **HR (95%CI)** | **p-value** |
| **Risk of mortality**  **(1-year) (n,%)** | 9,518 (6.3) | 5,784 (6.5) | 0.95 (0.92 to 0.98) | 0.002 | 3,819 (6.3) | 3,637 (6.0) | 1.02 (0.98 to 1.06) | 0.12 |
| **Risk of EA**  **(1-year) (n,%)** | 24,447 (16.2) | 8,098 (9.1) | 1.84 (1.79 to 1.89 | <0.0001 | 8,607 (14.2) | 6,910 (11.4) | 1.29 (1.26 to 1.33) | <0.0001 |
| **AECOPD incidence**  **(1 year) (n,%)** | 8,762 (5.8) | 4,805 (5.4) | 1.03 (0.99 to 1.07) | 0.32 | 3,576 (5.9) | 3,394 (5.6) | 1.02 (0.95 to 1.09) | 0.23 |
| **Female patients** | **COPD-CVD and BB use**  **(n=103,438)** | **COPD-CVD and no-BB use**  **(n=64,124)** | **HR (95%CI)** | **p-value** | **COPD-CVD**  **and BB use**  **(n=45,336)** | **COPD-CVD**  **and no-BB use**  **(n=45,336)** | **HR (95%CI)** | **p-value** |
| **Risk of mortality**  **(1-year) (n,%)** | 6,103 (5.9) | 3,847 (6.0) | 0.99 (0.95 to 1.03) | 0.65 | 2,584 (5.7) | 2,539 (5.6) | 1.06 (0.98 to 1.14) | 0.25 |
| **Risk of EA**  **(1-year) (n,%)** | 18,619 (18.0) | 7,438 (11.6) | 1.75 (1.71 to 1.79) | <0.0001 | 7,390 (16.3) | 6,120 (13.5) | 1.28 (1.24 to 1.32) | <0.0001 |
| **AECOPD incidence**  **(1 year) (n,%)** | 6,807 (6.6) | 4,104 (6.4) | 1.03 (0.99 to 1.07) | 0.08 | 2,992 (6.6) | 2,947 (6.5) | 0.99 (0.96 to 1.02) | 0.24 |
| HR:Hazard Ratio, CI: Confidence Interval, EA: Emergency admission, COPD: Chronic obstructive pulmonary disease, AECOPD: acute exacerbation of COPD CVD: Cardiovascular disease, BB: Beta-blocker | | | | | | | | |
