# Supplementary material for: Outcomes of beta-blocker use in people living with chronic obstructive pulmonary disease and a co-existent beta-blocker indicated cardiovascular disease. Insights from a global federated network
Source: BMC Pulm Med. 2026 Mar 4;26:166. doi: 10.1186/s12890-026-04216-z (PMC13067551; doi:10.1186/s12890-026-04216-z)
Supplement: Supplementary file 7 — Supplementary Material 7. [file 12890_2026_4216_MOESM7_ESM.docx]

| **Supplementary table 7. Risk of 1-year mortality, emergency admission and acute exacerbations of COPD versus beta-blocker users and non-users based on age before and after propensity score matching** | | | | | | | | |
| --- | --- | --- | --- | --- | --- | --- | --- | --- |
| **Geriatric patients**  **(Age>=65 years)** | **Before Propensity Score Matching** | | | | **After Propensity Score Matching** | | | |
|  | **COPD-CVD and BB use**  **(n=234,058)** | **COPD-CVD and no-BB use**  **(n=150,395)** | **HR (95%CI)** | **p-value** | **COPD-CVD**  **and BB use**  **(n=100,437)** | **COPD-CVD**  **and no-BB use**  **(n=100,437)** | **HR (95%CI)** | **p-value** |
| **Risk of mortality**  **(1-year) (n,%)** | 15,214 (6.5) | 9,625 (6.4) | 1.01 (0.98 to 1.04) | 0.51 | 6,127 (6.1) | 6,328 (6.3) | 0.99 (0.96 to 1.02) | 0.16 |
| **Risk of EA**  **(1-year) (n,%)** | 36,981 (15.8) | 13,686 (9.1) | 1.85 (1.81 to 1.89) | <0.0001 | 14,653 (14.5) | 11,651 (11.6) | 1.31 (1.26 to 1.33) | <0.0001 |
| **AECOPD incidence**  **(1 year) (n,%)** | 14,278 (6.1) | 8,873 (5.9) | 1.03 (0.99 to 1.07) | 0.12 | 6,392 (6.3) | 6,048 (6.0) | 1.02 (0.93 to 1.11) | 0.34 |
| **Younger patients**  **(Age<65 years)** | **COPD-CVD and BB use**  **(n=33,242)** | **COPD-CVD and no-BB use**  **(n=13,361)** | **HR (95%CI)** | **p-value** | **COPD-CVD**  **and BB use**  **(n=10,954)** | **COPD-CVD**  **and no-BB use**  **(n=10,954)** | **HR (95%CI)** | **p-value** |
| **Risk of mortality**  **(1-year) (n,%)** | 1,263 (3.8) | 414 (3.1) | 1.20 (1.12 to 1.28) | <0.0001 | 394 (3.6) | 351 (3.2) | 1.07 (0.95 to 1.19) | 0.32 |
| **Risk of EA**  **(1-year) (n,%)** | 7,579 (22.8) | 1,897 (14.2) | 1.48 (1.42 to 1.54) | <0.0001 | 2,246 (20.5) | 1,994 (18.2) | 1.17 (1.10 to 1.24) | <0.0001 |
| **AECOPD incidence**  **(1 year) (n,%)** | 1,995 (6.0) | 828 (6.2) | 0.98 (0.91 to 1.05) | 0.36 | 723 (6.6) | 679 (6.2) | 1.03 (0.98 to 1.05); | 0.45 |
| HR:Hazard Ratio, CI: Confidence Interval, EA: Emergency admission, COPD: Chronic obstructive pulmonary disease, AECOPD: acute exacerbation of COPD HFrEF: Heart failure reduced ejection fraction, AMI: Acute myocardial infarction, AF: Atrial fibrillation, BB: Beta-blocker | | | | | | | | |
