# Supplementary figures and images for: Outcomes of beta-blocker use in people living with chronic obstructive pulmonary disease and a co-existent beta-blocker indicated cardiovascular disease. Insights from a global federated network
Source: BMC Pulm Med. 2026 Mar 4;26:166. doi: 10.1186/s12890-026-04216-z (PMC13067551; doi:10.1186/s12890-026-04216-z)

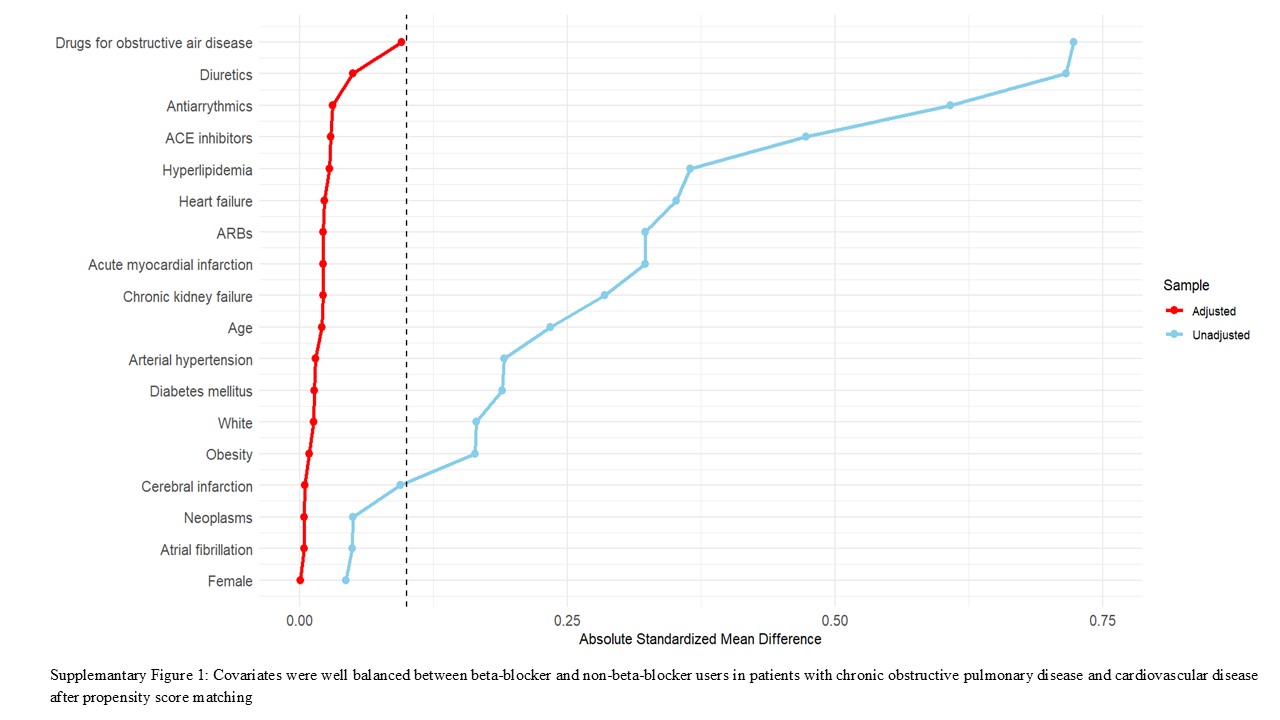

Supplement: Supplementary file 8 — Supplementary Material 8. [file 12890_2026_4216_MOESM8_ESM.jpg]
